# Effects of Methyl Mercury Chloride on Rat Hippocampus Structure

**DOI:** 10.1007/s12011-015-0492-3

**Published:** 2015-09-10

**Authors:** Jingwei Wu, Guangyuan Cheng, Zhiyan Lu, Mingyue Wang, Jianying Tian, Yongyi Bi

**Affiliations:** Department of Radiology, Zhongnan Hospital of Wuhan University, Wuhan, China; Department of Radiology, Hainan Provincial Nongken Hospital, Haikou, China; Department of Anatomy, Ningxia Medical University, Yinchuan, China; School of Public Health, Wuhan University, Wuhan, China

**Keywords:** Methyl mercury chloride (MMC), Cognitive dysfunction, Hippocampus

## Abstract

The objective of this study is to investigate the impacts of Methyl Mercury Chloride (MMC) on cognitive functions and ultrastructural changes of hippocampus in Sprague Dawley (SD) rats. Thirty healthy 20-day-old male SD rats weighing 30–40 g were randomly divided into three groups to receive daily injections. Two different dose levels were used: 4 mg/kg as high dose (H-MMC) and 2 mg/kg as low dose (L-MMC).The control group received 4 mg/kg saline solution (N-NaCl). After daily subcutaneous injection for 50 days, 6-day Morris water maze tests were used to assess the learning and memory functions of the rats. After a 5-day continuous training, spatial probe tests were conducted of times and paths crossing to the target quadrant on the 6th day. After the rats were euthanized, their hippocampus sections were stained with hematoxylin and eosin and analyzed under bothoptical microscope and electron microscope. The time H-MMC group spent in finding platform was significantly longer as compared toN-NaCl group on day 2 to day 5 and L-MMC group on day 4 to day 5. The number of crossing times of H-MMC group to the target quadrant was 0.63 ± 0.74, which is much lower than C-NaCl group (3.13 ± 1.56) with *P* value <0.05. No statistically significant difference in crossing times was found between L-MMC and C-NaCl groups. For H-MMC group, decreasing number of neurons and disorganized nerve cells were examined under light microscope. Swelling and dissolution of Golgi complex were examined under electron microscope, along with endoplasmic reticulum expansion and cytoplasmic edema. Mild cytoplasmic edema was found in L-MMC group. MMC can cause cognitive impairment in terms of learning and memory in SD rats. Additionally, it can also cause changes in the ultrastructure of neurons and morphological changes in the hippocampus, causing significant damage.

Methyl mercury chloride (MMC) is an environmental pollutant. It can be easily absorbed through the digestive tract and deposits in the kidney, liver, and other organs. Methyl mercury has a high thiol affinity and effective enterohepatic circulation. It is not easily discharged and accumulated in the body, especially in brain cells, causing significant damage to the nervous system. Studies have shown that MeHg can enter the brain directly by crossing the blood-brain barrier and accumulate in the hippocampus [1–3]. Recent studies have shown that when MeHg caused damage to the hippocampus—a brain area critical to memory formation—the learning and memory functions of hippocampus was damaged [4, 5].

This experiment was conducted on 20-day-old rats, exposed to methyl mercury for 50 days. Then the rats underwent Morris water maze (MWM) test to evaluate spatial learning, memory functions, and other cognitive functions. Hematoxylin-eosin staining (HE staining) and electron microscopy were also used to observe the changes of morphology and ultrastructure in hippocampal neuron, respectively.

## Materials and Methods

### Step SD Rats Infected Methyl Mercury Chloride

Weighing between 30–40 grams, taken from Wuhan University ABSL-III lab, 30 SPF level male SD (Sprague Dawley) rats were used in this experiment. They were randomly divided into three groups: H-MMC (high-dose group), L-MMC (low-dose group), and N-NaCl (control group, normal saline group). Each group was subjected to 100 mg MMC (Merck, Germany), purchased from Chengdu Ai Keda pharmaceutical company. After 1 week, subcutaneous injection under the neck was done on all the rats. H-MMC group has daily injections of 4 mg/kg MMC. L-MMC group has daily injections of 2 mg/kg. Control group was subjected to 0.9 % saline 4 mg/kg. Injections were carried out for about 50 days. Because of skin damage caused by methyl mercury, two rats in high-dose group were dead due to neck skin ulceration and infection.

### Morris Water Maze Test

One week would be spent in Morris water maze test, after the rat dementia model was completed. Three groups were on spatial learning, cognitive, and behavioral function test. Reference standards were the Charles V Voorhees & Michael T Williams method of MWM spatial learning in rats and cognitive-behavioral function tests. It included place navigation and space exploration experiment. Twenty-four rats were chosen to participate in this test, because some did not meet test conditions. Each of the three groups had eight rats to ensure equality and statistical significance.

#### Place Navigation

This test lasted 5 days, and SD rats’ spatial learning and memory were tested. Using smart image acquisition software, Morris water maze pool was evenly divided into four quadrants. A point was selected in each quadrant for entering and was marked as A, B, C, and D. Each subject was trained once in each quadrant, totally for four times a day for 5 days. Escape latency, time elapsed for the rats to find platform and escape from the water, was observed and recorded on a computer after training. If the rats found the platform within 60 s, the results were recorded as escape latency. If the rats were not able to find the platform within 60 s, the rats were guided and they stayed on the platform to familiarize for 15 s. The training interval for each rat was once per hour.

#### Spatial Probe Test

Rats’ memories of the original platforms were tested. The platform was removed on the 6th day. A new entry point E was selected. Rats entered the water from point E; they were observed and recorded in 30 seconds of the crossing number of the original position. Each rat was tested only once.

### Materials of Brain Tissue and Fixed

Six hippocampal cerebral cortex tissues were selected in each group to be stained and imaged. Morphological changes in rats’ hippocampal neurons were observed under optical microscope. The rest of the two hippocampal tissues were completely stripped, fixed in 2 % glutaraldehyde, and cut into 1 mm^3^ sections for imaging.

### Statistical Analysis Data

Statistical software SPSS 17.0 was used to analyze the escape latency of three groups of rats. *T* test was administrated to all respondents to compare the mean values between each group on different days. The analysis of variance (ANOVA) method for and optimized block design was conducted for this study.

## Results

### Results of Morris Water Maze Escape Latency Test

Three groups of rats were placed into water through the entry point at each quadrant—marking as A, B, C, and D. Each rat’s escape latency was recorded. After 5 days of intensive training, all rats shortened their escape latency day by day.

**Fig. 1:**
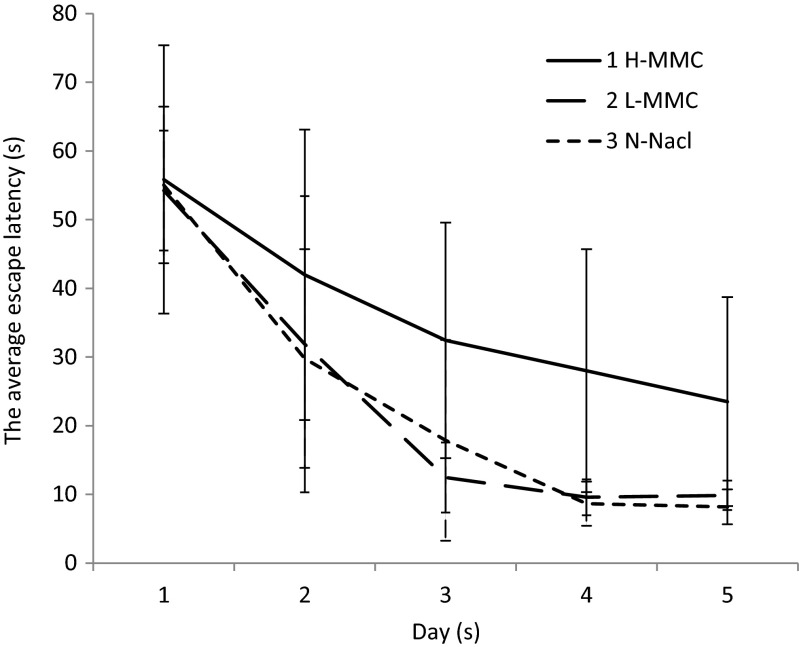
The effects of MMC on escape latency from Morris water maze test conducted over a period of 5 days

The mean values of daily escape latency among each group were compared by analysis of variance (ANOVA) method for and optimized block design. The mean differences between H-MMC and L-MMC, as well as H-MMC and N-NaCl were both statistically significant (*P* < 0.05). No statistically significant difference was found between L-MMC and N-NaCl (*P* > 0.05) (Table 1 and Fig. 1).

**Table 1:** Compared average escape latency (mean ± standard error)

Date	H-MMC (*n* = 8)	L-MMC (*n* = 8)	N-Nacl (*n* = 8)
Day 1	55.84 ± 19.53	54.23 ± 8.73	55.05 ± 11.40
Day 2	41.98 ± 21.14^a^	31.86 ± 21.55	29.78 ± 15.91
Day 3	32.43 ± 17.16^a^	22.47 ± 5.10	17.87 ± 14.60
Day 4	28.01 ± 17.68^a, b^	13.58 ± 2.62	10.64 ± 3.21
Day 5	23.50 ± 15.21^a, b^	9.86 ± 2.14	8.19 ± 2.53

^a^Statistically significant difference between H-MMC and N-NaCl

^b^Statistically significant mean difference between H-MMC and L-NaCl

### Results of Spatial Probe Test

**Fig. 2:**
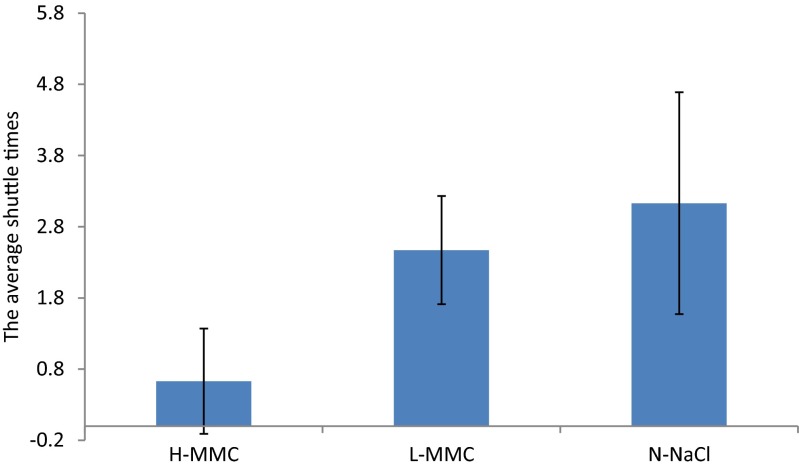
The shuttling times in the spatial probe test. The number of shuttling times of H-MMC group was statistically lower as compared to the L-MMC and N-NaCl groups

The number of times rats crossing the location of original platform (4th quadrant) with 30 s indicated the learning and memory ability through spatial probe test. The number of times that H-MMC group rats crossed the target quadrant containing the platform was (0.63 ± 0.74), which was statistically lower than the L-MMC group (2.47 ± 0.76) and the N-NaCl (3.13 ± 1.56) group. The difference between L-MMC and N-NaCl groups was not statistically significant (Table 2 and Fig. 2).

**Table 2:** Comparison of shuttling frequency (mean ± standard error)

Group	*n*	The shuttle times
H-MMC	8	0.63 ± 0.74^a^
L-MMC	8	2.47 ± 0.76
N-Nacl	8	3.13 ± 1.56

^a^Statistically significant difference between H-MMC and N-NaCl

### Results in Pathology Examination

**Fig. 3:**
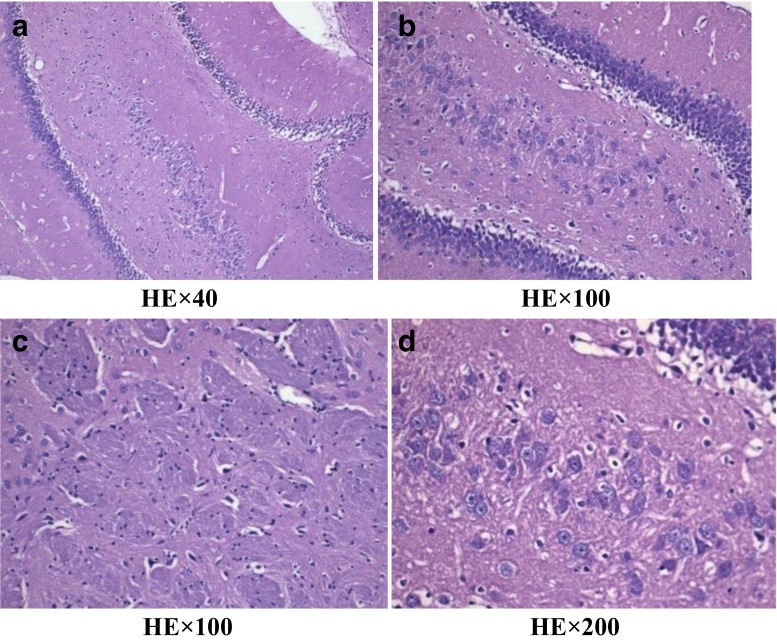
Effect of different concentrations of methyl mercury chloride (MeHgCl) on hippocampal injury in adult rats (hippocampus C1 area). Figure 3 in the high-dose MeHgCl group, a small number of hippocampal neurons appeared disorderly

**Fig. 4:**
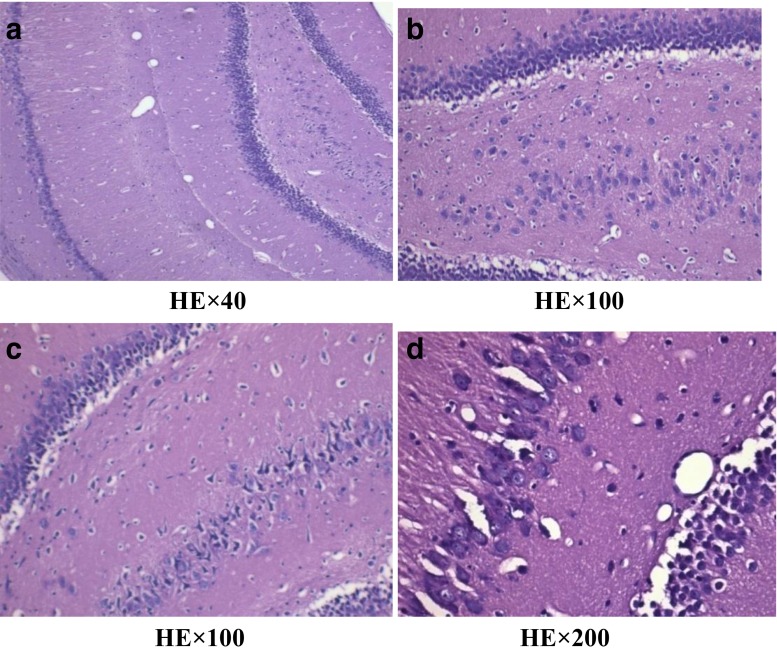
In the low-dose MeHgCl group, hippocampal nerve cells were tightly arranged and had a regular morphology and intact membrane

**Fig. 5:**
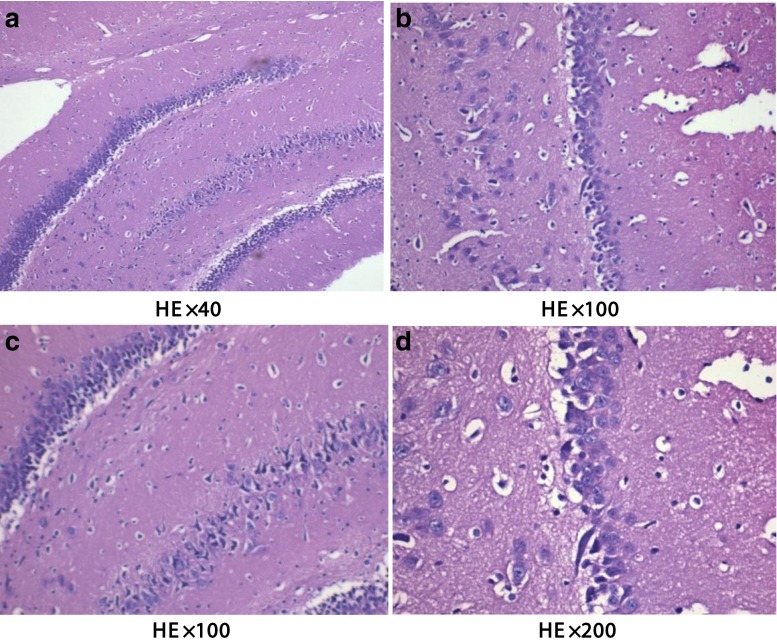
In the control group, the nerve cells in the hippocampus showed no obvious changes

Under the light microscope, we found the decrease in the number of hippocampal neurons and the disorganization of nerve cells for H-MMC group and were examined by HE stain (Fig.  3a–d). In contrast, L-MMC and N-NaCl group showed hippocampal neurons with regular shape and complete cell membrane (Figs. 4a–d and 5a–d).

**Fig. 6:**
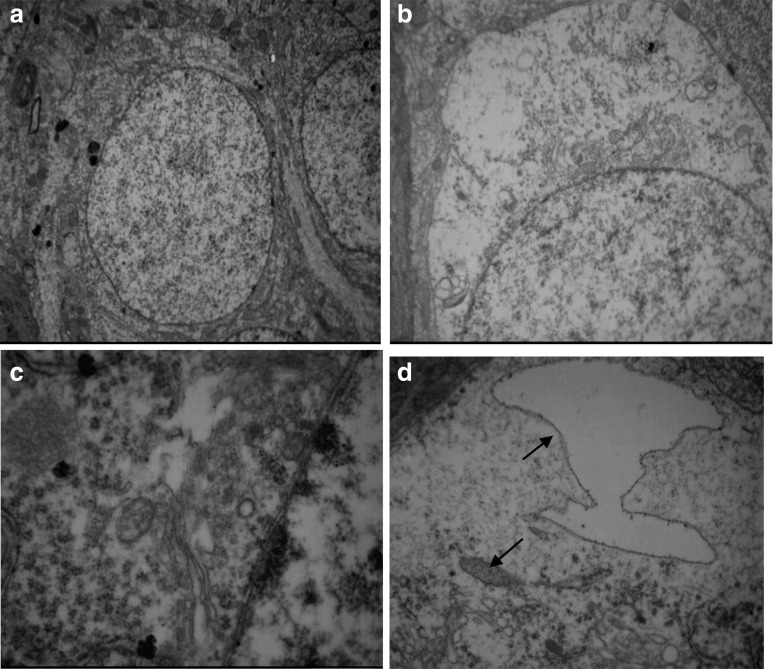
Electron microscope images of H-MMC. Effect of different concentrations of methyl mercury chloride (MeHgCl) on the ultrastructure of hippocampal tissue in adult rats. (Transmission electron microscopy, A, ×40,000). **a** Hippocampal nerve cells arranged neatly and coated complete. **b** Cells in the interstitial edema. **c** Endoplasmic reticulum expansion into the pool. **d** Golgi complex membrane dissolved, and part of Golgi complex swelling. (↑)

**Fig. 7:**
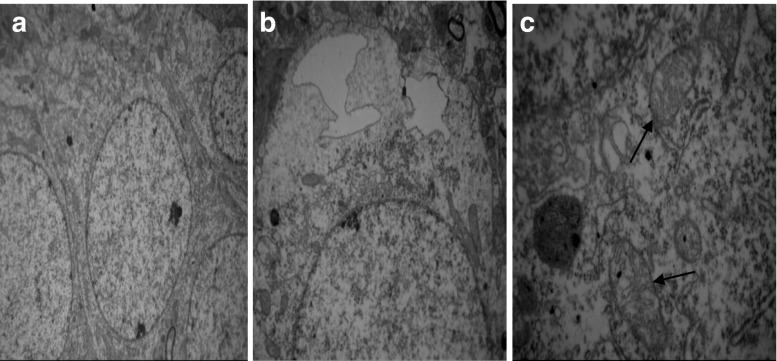
Electron microscope images of L-MMC. (Transmission electron microscopy, A, ×40,000). **a** Hippocampal nerve cells arranged neatly and coated complete. **b** No obvious interstitial the cell edema. **c** Some mitochondria swelling (↑)

**Fig. 8:**
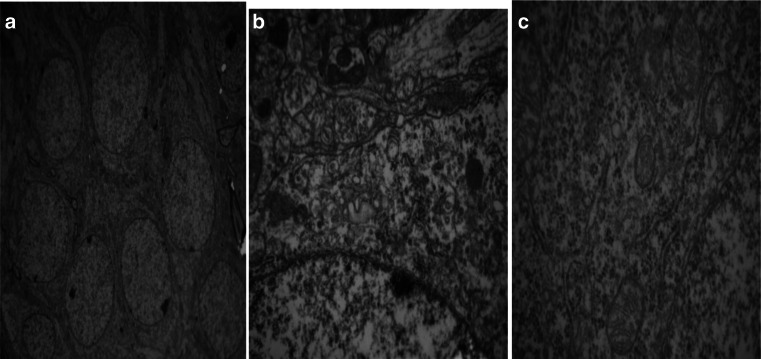
**a–c** Electron microscope images of N-Nacl. (Transmission electron microscopy, A, ×40,000). **a** hippocampal nerve cells arranged neatly and coated complete. **b** No obvious interstitial the cell edema. **c** Mitochondria, endoplasmic reticulum, and golgi complex organelles not seen obvious abnormity

Under electron microscope, for H-MMC group, swelling and dissolution of Golgi complex were observed, along with endoplasmic reticulum expansion, mitochondria hypertrophy, and cytoplasmic edema (Fig. 6a–d). There were obvious signs of endoplasmic reticulum expansion and mild cytoplasmic edema in L-MMC group rats (Fig. 7a–c). Both of the MMC injected groups of rats showed organized hippocampal neurons, complete cell membrane, and normal mitochondria. No *anomalous* hippocampal dentate gyrus cells were observed in N-NaCl group (Fig. 8a–c).

## Discussion

### Methyl Mercury on Hippocampal Nerve Damage

Methyl mercury, an organic mercury compound, composed of carbon atoms and mercury ions formation, is a highly toxic compound. Methyl mercury enters the body, completely absorbed by the intestinal tract, deposits in vital organs such as liver, kidney, and brain [6]. Methyl mercury is converted to methyl mercury chloride in the stomach. Because the human brain is rich in lipid and is also a major target organ of methyl mercury, the methyl mercury can enter the brain directly crossing the blood-brain barrier. Past literatures have shown that the hippocampus of animals has the highest concentration of mercury [1, 4]. Pregnant women and children are especially susceptible and sensitive to methyl mercury exposure. Not only can methyl mercury pass through maternal milk secretion in vitro, but also it can go through placental barrier without resistance. Hence, methyl mercury can be directly absorbed through the placenta, causing fetal harm even at low dosage, at the same time having minimal damage on pregnant women themselves. However, long-term exposure to methyl mercury can result in miscarriage, stillbirth, and fetal malformation, particularly damage to the developing nervous system [7].

### Cognitive Function Test by Morris Water Maze

After experimenting three groups of rats through the Morris water maze test came to completion, the average escape periods of infected rats on training days were significantly prolonged on day 2–5 when compared to that of the control group, especially in the high-dosage group. And the difference was statistically significant. However, there was no significance in the comparison between L-MMC and N-NaCl. On day 6, there was statistical significance in spatial learning between H-MMC and N-NaCl but none between L-MMC and N-NaCl. This finding is similar to that of the paper published by Miyamoto [8]. Miyamoto’s report stated that rats, which were 16 days old, suffered the most hippocampal and cortex injury than any other age. Kakita [9] and Goulet [10] conducted Morris water maze test on lactating and pregnant rats and found that athletic abilities, spatial, and learning abilities are damaged in their offspring. Their findings were further corroborated by Kantarci, Knopman, and Dickson’s [11] results. Sakamoto [12] and others’ studies suggested that due to rapid increase in brain volumes, offspring mercury concentration in the brain and body will decrease and that brain tissue damage can be gradually reverted. But this recovery does not undo the damage already sustained, especially in terms of learning and memory in the adults. Morris water maze test results indicated that a certain dose of methyl mercury neurotoxicity can damage hippocampal neurons and cognitive function.

### Pathologic Changes

In the H-MMC group, there was a reduction in the number of hippocampal neurons, as well as disorder in nerve cells. However, in the L-MMC group, hippocampal neurons were arranged in neat rows and no morphological anomaly was found. In H-MMC group, there were hippocampal dentate gyrus Golgi complex hypertrophy, edema, Golgi complex membrane lysis, expansion the endoplasmic reticulum, mitochondrial hypertrophy, and interstitial cell edema. Beer, Mielke, Zipp, Zimmermann, and Herdegen’s [13] study found that under electron microscope, there were ultrastructural changes and mitochondria disintegration in the hippocampus. Mercury exposure, to a certain extent, affected the normal development of the cerebellum and hippocampus. Stern’s [14] study has confirmed that hippocampal tissue gradually recovers after withdrawal of mercury exposure. Guo Li grew neuroglia in vitro and added MMC. The culture, under electron microscope, was found to have mitochondria appear in clusters. There were also change in vacuoles and swelling in the endoplasmic reticulum. In Zhang Hong’s experiments, rats were subjected to low dosage (1 mg/kg). Electron microscope found reduced cell numbers, cell membrane rupture, and necrosis. We did not find such results in our study, but we will continue our research.

Neurons in the central nervous system are equally sensitive to NMDA receptor; this may due to the increase in expression of NR2C subtype by exposure to methylmercury [15]. Methyl mercury can influence the expression of several subtypes of NMDA receptor. In addition to causing structural and functional changes in NMDA receptors, methyl mercury can also affect neuronal synaptic plasticity and the development of the central nervous system. This result is consistent with the findings of M Baraldi and P Zanoli [16, 17]. Their report stated that methyl mercury can cause changes in NR2 and NMDA receptor expression.

### Limitations of this Experiment

Due to individual differences in the process of exposing rats to methyl mercury, some rats developed infection, skin ulcers, and ultimately died. Some rats have extremely low tolerance to methyl mercury such that low dosage can be fatal. Some rats developed hemiplegia due to subcutaneous injections. Because of that, these rats were excluded from the water maze test.

## Conclusion

Rats infected with methyl mercury chloride showed damaged hippocampal ultrastructure. Imaging on optical microscope showed there was a reduction in the number of hippocampal neurons. Electron microscopy showed evident abnormalities in dentate gyrus Golgi complex, endoplasmic reticulum, and mitochondria in high-dose group, while low-dose group dentate gyrus only see the endoplasmic reticulum and cell interstitial edema. Through Morris water maze test, rats in high-dose group showed significant decrease in memory and cognitive functions.
